# Dislocated dental bridge covering the larynx: usefulness of tracheal tube guides under video-assisted laryngoscopy for induction of general anesthesia, thus avoiding tracheostomy

**DOI:** 10.1186/1746-160X-10-23

**Published:** 2014-06-11

**Authors:** Hiroshi Hidaka, Takahiro Suzuki, Hiroaki Toyama, Shin Kurosawa, Kazuhiro Nomura, Yukio Katori

**Affiliations:** 1 Departments of Otolaryngology Head and Neck Surgery, Tohoku University Graduate School of Medicine, 1-1 Seiryomachi, Aoba-ku, Sendai 980-8575, Japan; 2 Department of Anaesthesiology and Intensive Care Medicine, Tohoku University Graduate School of Medicine, 1-1 Seiryomachi, Aoba-ku, Sendai 980-8575, Japan

**Keywords:** Dental bridge, Foreign body, Dislodgement, Tube introducer, Tracheostomy

## Abstract

**Background:**

To describe a case with dislodgement of dental bridge with clasps covering the vocal cords, in a patient who was successfully intubated using tube exchanger under video-assisted laryngoscopy.

**Study design, methods:**

Clinical case record with a video clip.

**Setting:**

University hospital.

**Case presentation:**

A 83-year-old woman presented with dislodgement of her dental bridge whilst eating. Laryngoscopy revealed a foreign body almost entirely covering the vocal cords, with the clasps of the dislodged partial denture piercing the pharyngeal wall. Before induction of general anesthesia, a tracheal tube introducer combined with video-assisted laryngoscopy was introduced into the trachea in the awake condition, followed by successful endotracheal intubation. Thereafter, the dislodged denture was extracted via the oral cavity.

**Conclusions:**

Tracheal tube introducers combined with video-assisted laryngoscopy appear to be useful for airway management, decreasing the number of avoidable tracheostomies performed.

## Background

Aspiration or ingestion of dentures with a fixed bridge potentially results in serious morbidity because, in addition to the edge of the bridge traumatizing the lining mucosa, it can cause airway obstruction [1-3]. Supra- or subglottic denture-related foreign bodies of dentures, while relatively uncommon, have been reported to present with acute life-threatening upper airway obstruction, necessitate tracheotomy [4]. We herein report a case of a dislodged dental bridge with clasps covering the vocal cords, in a patient who was successfully intubated using a tube introducer under video-assisted laryngoscopy, followed by removal of the dentures under general anesthesia.

## Case presentation

An 83-year-old woman presented to our department after dislodgement of her dental bridge whilst eating. The patient was suffering from dementia due to Alzeimer’s disease, and had been admitted to a hospital for long-term care. A doctor at that hospital tried to remove the dentures, but failed.

**Figure 1:**
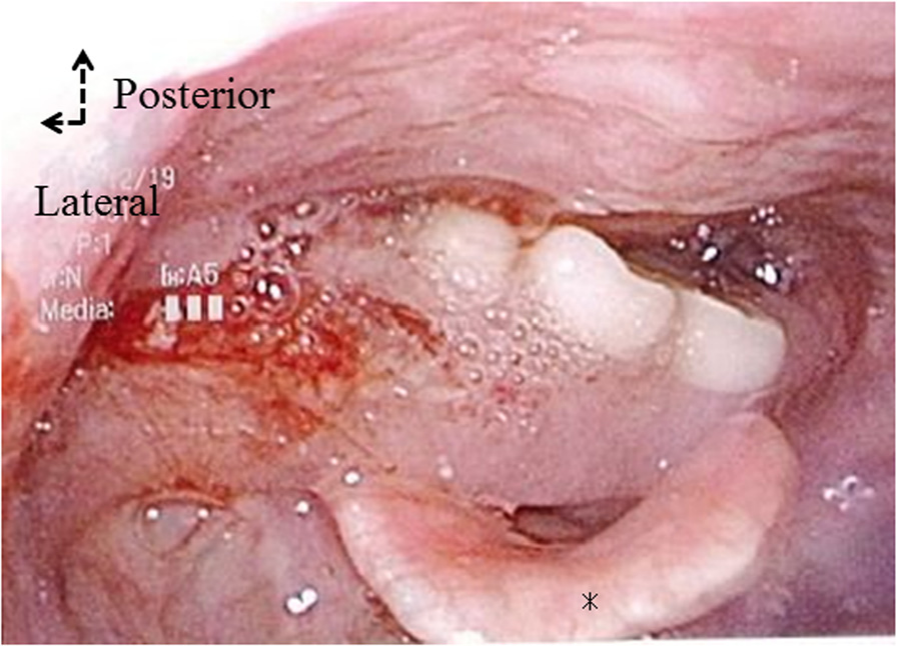
Fiberscopic view of the pharyngo-larynx showing a denture, obstructing the larynx.

**Figure 2:**
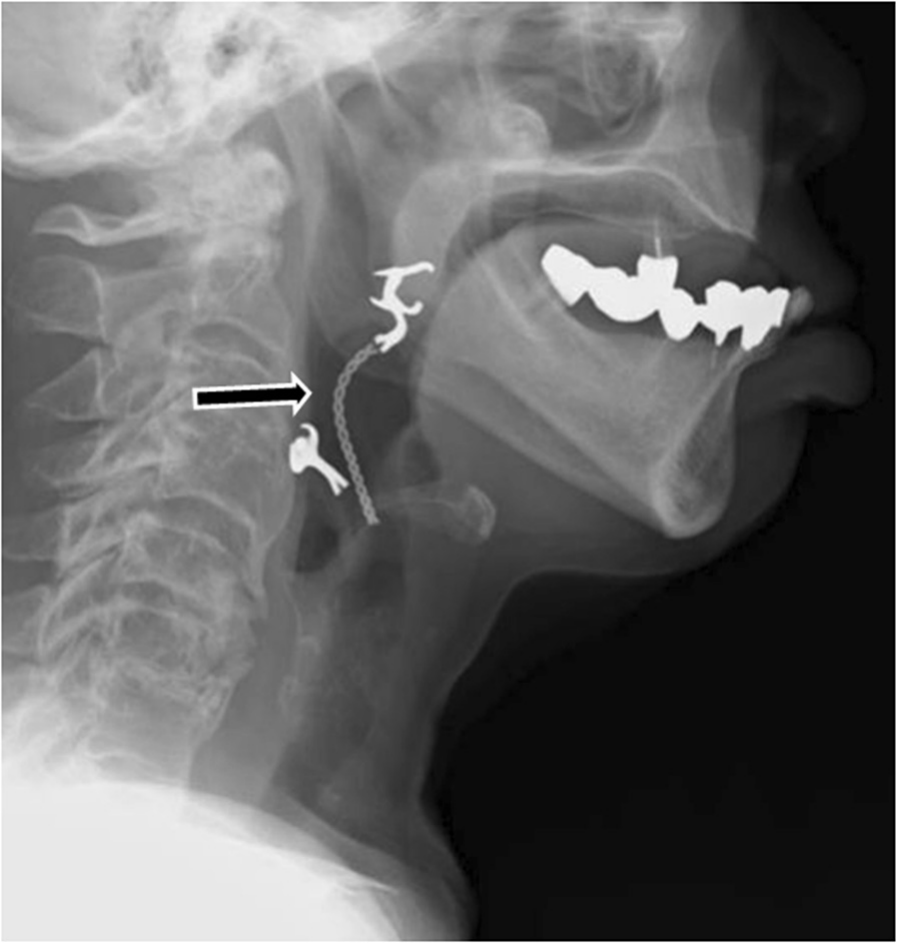
Radiograph of the neck showing a denture lodged in the pharygo-larynx (arrow).

The patient was not cyanotic, and did not complain of dyspnea, but was able to communicate with whispers. Her oxygen saturation was 95%. On physical examination, there was no stridor or suprasternal retraction. Flexible laryngoscopy revealed a foreign body in the pharyngolarynx, almost entirely covering of the supraglottic region other than the epiglottis. It was not possible to visualize portion of the vocal cords (Figure 1 and Additional file 1). Plain cervical spine radiographs showed the missing dental bridge, projecting from the epipharynx downward to the larynx (Figure 2). The foreign body seemed not easily removable with Magill forceps, because the clasps of the partial denture had pierced the pharyngeal mucosa.

**Figure 3:**
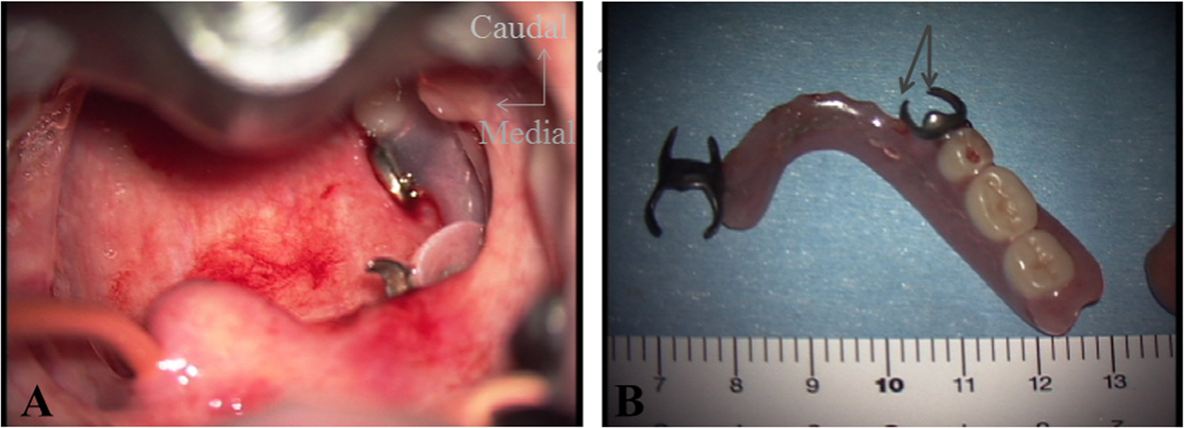
**Operative findings. (A)** A denture lodged in the pharynx. Note the lower clasp of the denture piercing the posterior wall of the pharyngeal mucos a. **(B)** The extracted denture. The arrow points to the lower clasp that pierced the posterior wall of the pharynx.

She was, thus, transported to the operating room for removal of the foreign body under general anesthesia. Before surgery, the first author consulted with the anesthesiologists regarding the findings of physical examination of the airway, and several airway management strategies were prepared, including tube exchangers, fiberscopic intubation, and direct laryngoscopes. We also kep a tracheostomy kit ready, in case of failure to intubate. After preoxygenation via a face mask, topical lidocaine was administered into the oropharynx. Following awake video-assisted laryngoscopy with a Macintosh type blade (GlideScope®, Verathon Medical Inc., Canada) revealed the epiglottis, a tracheal tube introducer (15 French, size; Portex) was introduced into the trachea, followed by successfully endotratrachial intubation (endotracheal tube; I.D. 6.5 mm), achieved by railroading the tube over the bougie. General anesthesia was thereafter induced.After insertion of a self-retaining mouth gag for the use of tonsillectomy, the pharynx was observed using an operating microscope. The upper part of the clasp of the removal dentures was seen to be attached to the posterior wall of the soft palate, while the lower clasp had pierced the mesopharyngeal mucosa (Figure 3A). By detaching the piercing clasp from the mucosa, the denture, comprising two unit bridges, was extracted via the oral cavity (Figure 3B).

Postoperative chest and neck X-rays revealed no dentures or pneumonia. Consultation with a dentist revealed that the offending dentures were probably too loose for her lower gingiva, and hence, replacement with new dentures was recommended. The patient had no further problems, recovering uneventfully without dyspnea or dysphagia, and was transferred back to the previous long-term care hospital.

## Discussion

Foreign bodies are common causes of choking and respiratory tract obstruction. Aspiration of a dental prosthesis with a fixed bridge represents serious morbidity, because the edge of the bridge may traumatize the lining mucosa, in addition to potentially causing airway obstruction [1-3]. Previous studies reported that general anesthesia is more frequently required for removal of dentures than for other types of foreign bodies since their safe removal mandatorily requires evaluation of their size and shape [5]. Our patient’s dementia-related uncooperative state also contributed to our decision to remove the denture under general anesthesia.

To the best of our knowledge, ours is the first report addressing the usefulness of tube guides for intubation in cases with difficult airways due to dislodged dentures, thus obviating the needs for tracheostomy. Physical examination prior to intubation and anesthetic induction facilitates detection of a difficult airway [6]. Our case reinforces the importance of collaboration between the surgeon and anesthetist in cases with airway foreign bodies, to enable appropriate planning for the induction of general anesthesia. Since preoperative examination revealed that the dentures almost entirely covered our patient’s larynx, we were prepared for the possible need for tracheostomy in case of failure to incubate. However, the decision to perform a tracheotomy should be made with much care, as the procedure is not without potential complications particularly in uncooperative [7]. Moreover, disturbance of the normal mechanism of swallowing is sometimes encountered after tracheostomy, presumably resulting from desensitization of the larynx after diversion of the air-passage or fixation of the larynx [8]. In the present case, the dental bridge almost entirely covered the supraglottic region other than the epiglottis, with limited visualization of the vocal cords. However, she was not suffering from dyspnea, presumably because some air space would exist vertically between the dental bridge and the vocal cords. Even though the airway of our patient was almost likely to be at risk, less invasive procedures without tracheostomy would be a useful option in such a patient without complete airway obstruction.

Tracheal tube (bougie) introducers, which have been marketed since the early 1970s, differs from previous introducers in their greater length (60 cm), angled tip and the combination of flexibility and malleability [9]. They are readily available and the technique of their use combines simplicity of operation with a high success rate [6,9]. The intubation method, which included combination of the bougie introducer with the Macintosh laryngoscope, has been the most widely used technique in the UK. However, the usefulness of this technique with glidscope has not been previously described. Specifically, the glidescope with bougie technique in the present case was most likely useful because it allowed direct visualization of the supraglottic larynx with an eye of the bougie through a partially obstructed laryngeal inlet, partly because of the bougie’s narrow diameter as compared with tracheal tube. Furthermore, the ability to visualize movement of the larynx in relation to the foreign body with a videolaryngoscope would make these techniques more useful than those using a fiberoptic bronchoscope for tracheal intubation. Again, this technique would be advantageous over the intubation using rigid bronchoscopy because of being less burdened to the patient in awake condition [10]. After the induction of general anesthesia, we could safely remove the dentures by detaching the piercing clasp from the mucosa.

Aspiration of dental restorations or teeth themselves is most likely to occur in relation with dental treatment, ethanol intoxication or maxillofacial trauma [10]. Conversely, dislodgement of dentures might be attributed to loose dentures, as in the current case. Previous report mentioned that dentures need re-aligning or replacement with the passage of time and ongoing gum remodeling [3]. Loose dentures are relatively easy to dislodge and can cause life threatening airway obstruction [4]. Despite a large number of older people using dentures, assessment of loose-fitting dentures is not common practice, and is largely left to the discretion of patients and their dentists [4]. Particularly in older patients suffering from dementia, enquiries about loose-fitting dentures should be an integral part of comprehensive geriatric assessment, to prevent their dentures becoming dislodged.

## Conclusion

Aspiration or dislodgement of dentures with a fixed bridge has the potential to cause serious morbidity because the edge of the bridge may traumatize the lining mucosa in addition to causing airway obstruction. Glidescope with bougie technique using tube introducers are likely to be useful in such cases, because it allowed direct visualization of the supraglottic larynx with an eye of the bougie through a partially obstructed laryngeal inlet. This technique may decrease the number of avoidable tracheostomies performed.

## Consent

Written informed consent was obtained from the patient for publication of this Case report and any accompanying images. A copy of the written consent is available for review by the Editor-in-Chief of this journal. The review board of Tohoku University Hospital approved publication of this case report.

## Competing interests

The authors declare that they have no competing interests.

## Authors’ contributions

HH and TS, surgery, and manuscript editing; HT, anesthetic management, and manuscript editing; and SK, clinical consultation and editing of manuscript; KN and YK, revised manuscript editing. All authors read and approved the final manuscript.

## Supplementary Material

Additional file 1 Video clip of Figure S1, showing a fiberscopic view of the pharyngo-larynx, with the denture interfering with visualization of the vocal cords. Click here for file
